# Curcumin Reduces Amyloid Fibrillation of Prion Protein and Decreases Reactive Oxidative Stress

**DOI:** 10.3390/pathogens2030506

**Published:** 2013-07-25

**Authors:** Chi-Fen Lin, Kun-Hua Yu, Cheng-Ping Jheng, Raymond Chung, Cheng-I Lee

**Affiliations:** 1 Department of Life Science, Institute of Molecular Biology and Institute of Biomedical Science, College of Science, National Chung Cheng University, Min-Hsiung, Chia-Yi, Taiwan 621, China; E-Mails: fen2170@yahoo.com.tw (C.-F.L); ykhuna@gmail.com (K.-H.Y.); q246855@yahoo.com.tw (C.-P.J.); 2 Department of Clinical Pathology, Buddhist Dalin Tzu Chi General Hospital, Chia-Yi, Taiwan 622, China; 3 Department of Chemistry and Biochemistry, Manhattan College, Riverdale, NY 10471, USA

**Keywords:** prion, amyloid, fibril, curcumin, apoptosis, ROS

## Abstract

Misfolding and aggregation into amyloids of the prion protein (PrP) is responsible for the development of fatal transmissible neurodegenerative diseases. Various studies on curcumin demonstrate promise for the prevention of Alzheimer’s disease and inhibition of PrP^res^ accumulation. To evaluate the effect of curcumin on amyloid fibrillation of prion protein, we first investigated the effect of curcumin on mouse prion protein (mPrP) in a cell-free system. Curcumin reduced the prion fibril formation significantly. Furthermore, we monitored the change in apoptosis and reactive oxygen species (ROS) level upon curcumin treatment in mouse neuroblastoma cells (N2a). Curcumin effectively rescues the cells from apoptosis and decreases the ROS level caused by subsequent co-incubation with prion amyloid fibrils. The assays in cell-free mPrP and in N2a cells of this work verified the promising effect of curcumin on the prevention of transmissible neurodegenerative diseases.

## 1. Introduction

Conformational diseases are characterized by structural conversion of proteins to alternative forms, which subsequently convert into protein fibrils. The accumulation of these protein fibrils as amyloid deposits in the brain is implicated in a number of neurodegenerative diseases, including Alzheimer’s disease, transmissible spongiform encephalopathies, Parkinson’s disease, among others [1,2]. Of particular interest are transmissible spongiform encephalopathies. 

Transmissible spongiform encephalopathies (TSE) are a group of fatal neurodegenerative diseases in mammals [1], diseases that include Creutzfeldt-Jakob disease, Bovine spongiform encephalopathy, Scrapie, Kuru, Gersmann-Sträussler-Scheinker syndrome and fatal familial insomnia. The aetiology of TSEs can be genetic, sporadic or infectious [3]. The cause of TSEs is believed to be misfolding of the cellular isoform of prion protein (PrP^c^), which is rich in α-helical structure, to the scrapie isoform of PrP (PrP^Sc^), which is rich in β-sheet structure. PrP^Sc^ aggregates to form stable, insoluble, and proteinase K-resistant fibrils. Accumulation of such prion fibrils in the brain is believed to cause neuronal cell death and onset of disease. However, the mechanism of neuronal cell death continues to be unclear. 

Many compounds have been identified as inhibitors of PrP^Sc^ formation, but none of these compounds are known to be safe or effective for use in humans and animals [4]. Recent efforts have identified curcumin as an inhibitor of prion fibril formation [4,5]. Curcumin is also considered as a powerful anti-inflammatory agent [6] involving toll-like receptors [7]. Curcumin, or 1,7-bis(4-hydroxy-3-methoxyphenyl)-1,6-heptadiene-3,5-dione, is the main yellow pigment derived from the rhizome of turmeric (*Curcuma longa*) and has been found to be capable of crossing the blood-brain barrier [8]. Caughey and co-workers have found that curcumin inhibits the accumulation of PrP^Sc^ in scrapie-infected neuroblastoma (scNB) cells [4]. Work done by Hafner-Bratkovic and co-workers has shown that curcumin binds only to non-native forms of PrP, thereby thwarting prion fibril formation without affecting native PrP [5]. Structurally, curcumin is similar to Congo red, which is a diazo dye known to bind to amyloid fibrils and, thus, used to stain amyloid plaques. Congo red has been shown to reduce the accumulation in scrapie-infected cells in the abnormal protease-resistant form (PrP^Sc^ or PrP^res^) [9] and to inhibit PrP^Sc^ formation in a cell-free system [10]. However, Congo red is toxic and a poor brain penetrant [8]. Due to its ability to inhibit PrP^Sc^ formation, its ability to cross the blood-brain barrier, and the fact that humans consume significant amounts of it without apparent toxicity, curcumin shows promise as an anti-TSE agent.

In addition to anti-amyloidogenic properties, curcumin is also known to have strong antioxidant properties. Other than amyloid plaque accumulation, mitochondrial dysfunction and high reactive oxygen species (ROS) levels are also characteristics of neurodegenerative diseases like Alzheimer’s disease, Parkinson’s disease, Huntington’s disease and amyotrophic lateral sclerosis [11,12,13]. In the context of TSEs, the production of ROS could be a cause of mitochondrial dysfunction resulting in neurodegeneration. Therefore, further studies of the antioxidant effects of curcumin on cells suffering from fibril-induced oxidative stress are warranted.

In the present study, the anti-amyloidogenic and antioxidant effects of curcumin on the behavior of recombinant murine PrP (mPrP) were investigated in a cell-free environment as well as in murine neuroblastoma (N2a) cells.

## 2. Results and Discussion

### 2.1. The Effect of Curcumin on the Cell-Free mPrP Amyloid Formation

The inhibition of PrP^c^-to-PrP^Sc^ conversion by curcumin has been demonstrated in hamster PrP [4] and in truncated human PrP [5]. However, the effect of curcumin on the structural conversion of mPrP is not known. This prompted us to initially investigate the effect of curcumin on cell-free conversion of mPrP.

**Figure 1:**
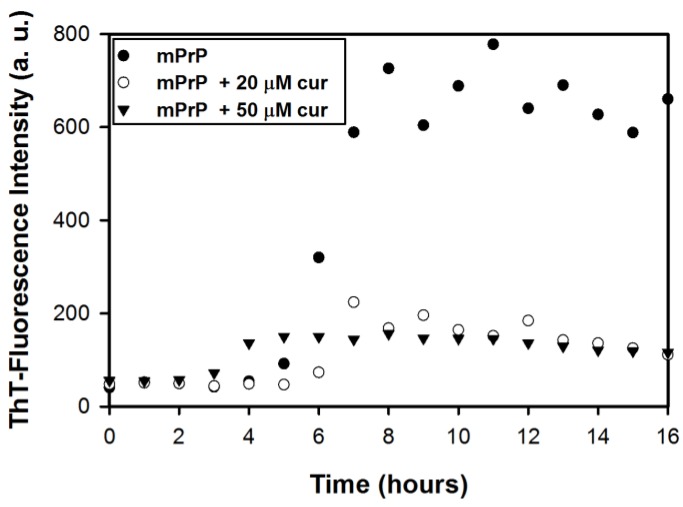
The thioflavin T (ThT)-fluorescence measurement of amyloid conversion from 20 μM mouse prion protein (mPrP) in the absence and in the presence of curcumin.

The effect of curcumin on the amyloid formation of mPrP was studied based on the kinetics of amyloid formation with and without curcumin. Incubation of recombinant mPrP in 2 M guanidine hydrochloride (GdnHCl) at 37 °C resulted in amyloid fibrils [14,15]. The formation of amyloid fibrils was determined by the fluorescence intensity of thioflavin T (ThT) at 480 nm arising from specific binding of ThT to cross β-sheet structures in amyloid fibrils. Formation of amyloid fibrils includes nucleation in the lag phase and subsequent elongation in the growth phase. As shown in Figure 1, in the absence of curcumin, the ThT-fluorescence reading of mPrP fibrils rose at the fifth hour and then increased significantly in the following three hours. Finally, the ThT-fluorescence intensity remained constant. This sigmoidal kinetic curve indicates that the duration of the lag phase and of the growth phase of mPrP fibril formation can be estimated as 5 h and 3 h, respectively. In contrast, the sigmoidal curve observed in the presence of curcumin (20 μM or 50 μM) exhibited a weak increase such that the ThT-fluorescence reading remained low. In the presence of 20 μM curcumin, the ThT-fluorescence intensity increased slightly after 5–6 h of reaction but did not increase significantly in the following incubation, indicating partial inhibition of the growth phase. This kinetic study confirms that curcumin reduces the formation of amyloid fibrils and that partial inhibition likely occurs at the growth phase. In the presence of 50 μM curcumin, the ThT-reading increased slightly after 3–4 h of incubation but remained low after the forth hour. This shortened lag phase implies the existence of an alternative pathway for nucleation, while the fibril elongation may have also been inhibited in the alternative pathway. The amyloid fibrils obtained in this experiment were further analyzed in the following experiments. 

**Figure 2:**
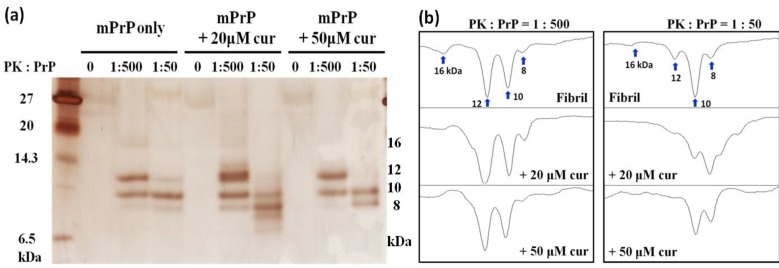
Proteinase K (PK) resistance of mPrP fibrils upon treatment of curcumin analyzed by (**a**) silver blotting in tricine-SDS-PAGE and (**b**) densitometric quantification of proteins below 20 kDa. Amyloid mPrP fibrils were grown without curcumin, with 20 μM and 50 μM curcumin (cur). Each fibril sample was treated with PK at the ratio of PK: mPrP = 0, 1:500 and 1:50. The approximate molecular weight of PK-resistant fragments were marked on the right side of the PAGE. The corresponding bands were labeled in the plot of densitometric analysis.

An important feature of PrP^Sc^ is the resistance to proteinase K (PK) activity. Therefore, we treated the mPrP fibrils with PK, and then analyzed the PK-resistant fragments with high-resolutiontricine-SDS-PAGE [16]. In tricine-SDS-PAGE, small protein fragments can be clearly resolved at ~12 and 10 kDa, as shown in Figure 2. At PK: PrP = 1: 500, the fragments with 12 and 10 kDa clearly existed in all the samples. A weak band at ~16 kDa was shown in curcumin-free samples but this band disappeared in curcumin-treated samples. When PK was increased such that PK: PrP =1:50, in the presence of 20 μM curcumin, the 12-kDa fragment was strongly weakened while a smaller fragment (~8 kDa) was observed. This pattern implies that the amyloid forms of PrP generated under curcumin treatment can be further digested. When 50 μM of curcumin was added, the 12-kDa fragment was not observed while the 10- and 8- kDa fragments were more pronounced than the same fragment in the absence of curcumin. This pattern indicates the presence of an alternative amyloid form generated under curcumin treatment. Further analysis of this alternative amyloid will be the subject of future study.

**Figure 3:**
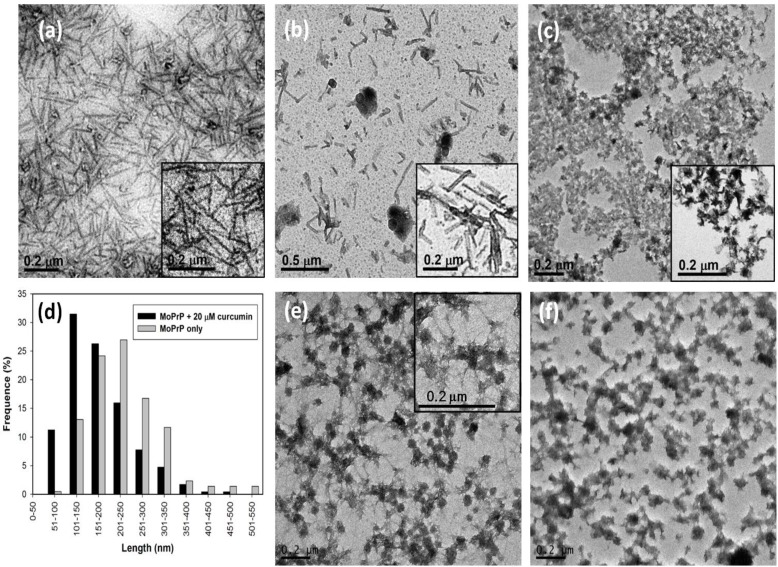
TEM images of mPrP fibrils grown (**a**) in the absence of curcumin; (**b**) in the presence of 20 μM and (**c**) 50 μM curcumin; (**d**) The analysis of length-distribution of fibrils grown in the absence of curcumin and in the presence of 20 μM curcumin. The grown mPrP fibrils were treated with (**e**) 20 μM and (**f**) 50 μM curcumin for 12 h.

As determined by X-ray fiber diffraction, amyloid fibrils associated with various diseases all appear as unbranched straight fibrils [17]. Thus, it is essential to observe the morphology of the fibrils converted in the presence of curcumin. TEM images of the fibrils under various conditions were analyzed. As illustrated in Figure 3a, the mPrP fibrils grown in the absence of curcumin are long and straight as observed in TEM. The straight mPrP fibrils were also observed in the presence of 20 μM curcumin as shown in Figure 3b, but the length of the fibrils were generally shorter than those grown in the absence of curcumin. The analysis of the fibril-length distribution plotted in Figure 3d indicates that the most populated fibrils were 201–250 nm long in the absence of curcumin, whereas the major population was shifted to 101–150 nm in the presence of 20 μM curcumin. The average length of mPrP fibrils grown in the absence of curcumin and in the presence of 20 μM curcumin is 238 and 174 nm, respectively. The diameter of each of these mPrP fibrils is about 25 nm. In the presence of 50 μM curcumin, the majority of the mPrP aggregated amorphously as shown in Figure 3c. Amyloid fibrils were rarely formed and the observed fibrils were curvy and short (~60 nm). These TEM images clearly suggest that curcumin inhibited the elongation of the mPrP amyloid fibrils along the long-axis of the fibrils. This is consistent with previous study indicating curcumin binds to the intermediates of PrP resulting inhibition of amyloid formation [5]. The grown mPrP fibrils were further treated with 20 μM and 50 μM curcumin for 12 h as shown in Figure 3e,f, respectively. Clearly, 20 μM curcumin impaired the morphology of fibrils such that the residual fibrils observed exhibited both straight morphology and amorphous aggregation. When the concentration of curcumin was increased to 50 μM, the amyloid-forms were mostly abolished. This phenomenon is consistent with a study of quercetin where quercetin induced the amorphous aggregation of mature insulin fibrils [18]. 

### 2.2. The Effect of Curcumin in Cells

To investigate the effect of curcumin in cells, we first confirmed the disruption of cell membranes in erythrocytes from mouse blood. Subsequently, cell viability, apoptosis and ROS level were all studied with various concentrations of curcumin. 

**Figure 4:**
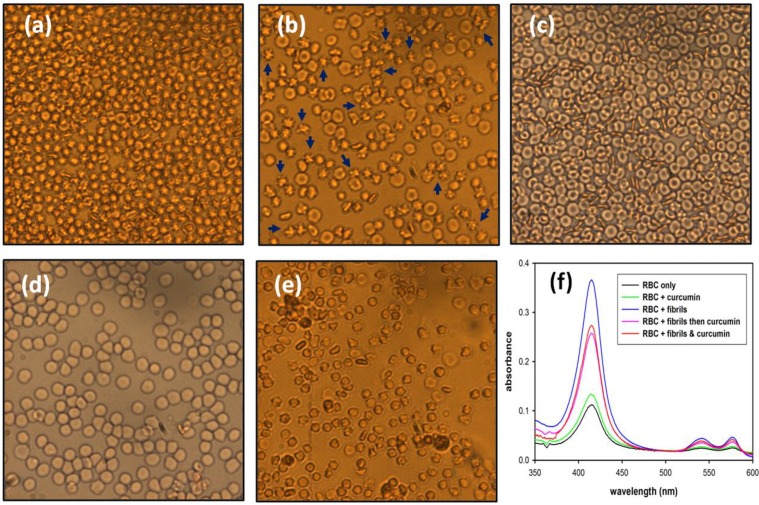
Hemolytic assay of mouse erythrocytes (**a**) before treatment; (**b**) after addition of 20 μM amyloid mPrP fibrils; (**c**) treated with curcumin without fibrils; (**d**) treated with curcumin and mPrP fibrils simultaneously and (**e**) treated with 5 μM curcumin prior to the addition of amyloid mPrP fibrils; (**f**) The amount of leaked oxy-hemoglobin monitored by absorption spectroscopy.

#### 2.2.1. Hemolysis of Mouse Blood

A previous study demonstrated that mouse erythrocytes can be damaged when they are co-incubated with insulin amyloid fibrils [18]. Mouse erythrocytes are typically oval and biconcave disks as shown in the image in Figure 4a. When the erythrocytes were co-incubated with mPrP fibrils in an isotonic buffer, cells of shriveled morphology were formed, as shown in Figure 4b, wherein the representative cells are indicated by blue arrows. On the other hand, the cells were rescued from membrane disruption by co-treatment or by pre-addition of 5 μM curcumin as shown in Figure 4d and e, while curcumin did not alter the cell membranes significantly as judged from Figure 4c,f. The mPrP fibrils caused cell membrane disruption resulting in the leakage of hemoglobin out of the erythrocytes. As confirmation, the absorption of oxy-hemoglobin at 415, 540 and 580 nm significantly increased in the isotonic buffer after the fibril treatment as compared in Figure 4f. Treatment of the erythrocytes with curcumin together with or prior to fibril incubation reduced membrane damage and lowered the amount of leaked oxy-hemoglobin (Figure 4f). Considering the TEM images of the stunted amyloid fibrils grown in the presence of curcumin and the attenuated hemolysis of the cells treated with curcumin, the inhibition of hemolysis is very likely due to impairment of fibrillar structure by curcumin. These results also suggest that amorphous aggregates have a weaker damaging effect on the cell membranes than do the mPrP amyloid fibrils formed without curcumin. 

A previous review of research on Alzheimer’s disease pointed out that oligomeric amyloid-β (Aβ) binds to membranes avidly and causes permeation [19]. The Aβ-induced membrane damage could be carried out by a combination of several mechanisms including carpeting, pore formation and the detergent effect [20]. In this case, the shriveled erythrocytes in Figure 4b are likely to be caused primarily by the fibril-induced pore formation, which results in leakage of the cytoplasmic matrix. The possibility of fibril-induced membrane disruption by carpeting and detergent effects would require further investigation.

#### 2.2.2. Viability, Apoptosis and ROS Level of N2a Cells

**Figure 5:**
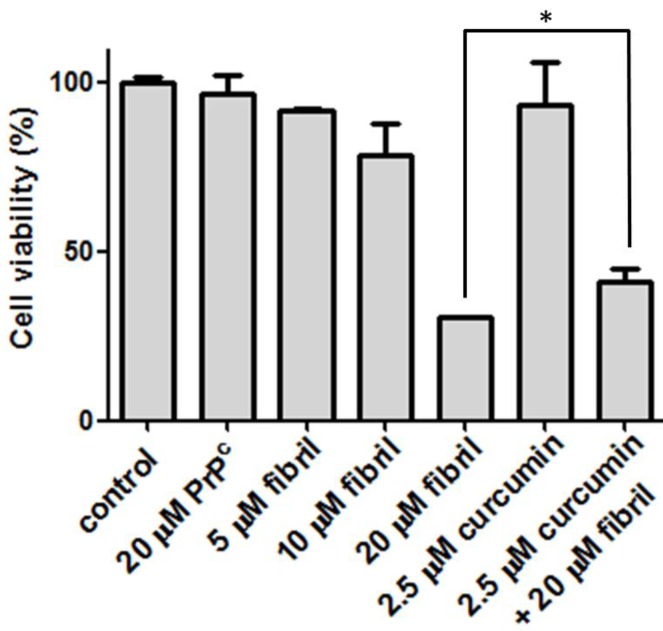
Viability of N2a cells before treatment (control), after addition of amyloid mPrP fibrils, and the treatment of curcumin prior addition of amyloid mPrP fibrils. To test the effect of PrP^C^, N2a cells were treated with 20 μM of mPrP. The statistical significance is represented by an asterisk (*p* < 0.05).

To determine the cytotoxicity of amyloid fibrils, our preliminary test is the viability of N2a cells after co-incubation with mPrP amyloid fibrils. The cell viability under various conditions compared in Figure 5 clearly indicates that the mPrP amyloid fibrils cause cell death in a dose-dependent manner, whereas mPrP is not toxic to the N2a cells. Considering that the curcumin level in human serum after oral intake with 4–8 g of curcumin is 0.4–3.6 μM [21], we lowered the curcumin dose to 2.5 μM in the following cell experiments. Treatment with 2.5 μM of curcumin increased the cell viability from ~30% to ~40%. This increase of ~33% cell viability suggests that cells can be rescued from cytotoxicity efficiently by treatment with curcumin prior to the co-incubation with amyloid fibrils. The daily intake of curcumin at 0.1–3 mg/kg body weight is suggested by the Joint FAO/WHO Expert Committee on Food Additives, 1996 [22]. According to the suggested diet information, a person with 60 kg can intake as much as 12 μM of curcumin per day. Experiments on patients consuming high doses of curcumin (up to 8000 mg daily) for three months have shown that curcumin does not carry detectable side effects [23]. As judged from our finding on healing from one-time treatment with low-dose curcumin, the acceptable daily intake level of curcumin can potentially abolish the toxicity caused by amyloid fibrils. A study comparing the population of patients with Alzheimer’s disease in the US and in the India reported relatively low incidence of Alzheimer’s disease in India [24]. Since it is one of the main ingredients in Indian cuisine, curcumin’s anti-amyloid effects might contribute to neuro-protection from Alzheimer’s disease. Unfortunately, two six-month clinical trials concerning the effects of curcumin on possible patients of Alzheimer’s disease reported no significant difference in cognitive function between placebo and curcumin groups [25]. Further clinical trials concerning the effect of curcumin on prevention and treatment of Alzheimer’s disease and TSEs are strongly suggested.

**Figure 6:**
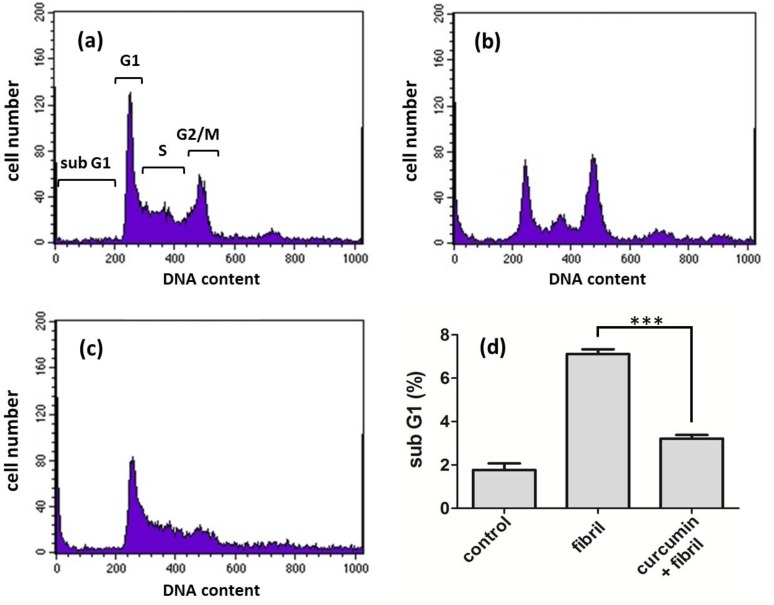
Apoptosis of N2a cells represented by the sub G1 population (**a**) before treatment; (**b**) after addition of 20 μM amyloid mPrP fibrils; and (**c**) the treatment of 2.5 μM curcumin prior to addition of 20 μM amyloid mPrP fibrils; (**d**) The apoptosis level compared by the sub G1 population. The statistical significance is represented by three asterisks (*p* < 0.001).

The decrease of cell viability upon exposure of cells to prion fibrils can be ascribed to apoptosis and necrosis. Apoptosis is the process of programmed cell death and we are interested in the fibril-induced defective apoptotic process. To further investigate the effect of curcumin at the cellular level, we studied the apoptosis of N2a cells treated with curcumin. The apoptosis assay provides information on biochemical events leading to cell death in the cell cycle. To detect cell apoptosis, the N2a cells were stained with fluorescent propidium iodide (PI) to recognize fragmented DNA in apoptosis and then analyzed by flow cytometry. The assignment of cell cycles including sub G1, G1, S, and G2/M phases are denoted in Figure 6a. Clearly, the fibril treatment increased the population of sub G1 and decreased the population of G1 and G2/M significantly as shown in Figure 6b. The population of sub G1 induced by fibril treatment was more than 3-fold compared to the population of sub G1 without fibril treatment, as shown in Figure 6d. This fibril-induced apoptosis was largely weakened when N2a cells were treated with curcumin prior to the fibril treatment (Figure 6c,d). In comparison with the result of cell viability shown in Figure 5, curcumin largely rescued cells from fibril-induced apoptosis but only slightly reduced fibril-induced necrosis. This result suggests that curcumin is involved in the cellular regulation and signal transduction. A previous study suggested that PrP is directly involved in the signaling cascade regulating as a Bcl-2-family member and that PrP is actually a neuroprotective agent against the major apoptotic Bax protein [26]. Therefore, the treatment of curcumin prior the addition of mPrP fibrils inhibits the formation of amyloid forms and, thus, preserves the neuroprotective role of mPrP.

**Figure 7:**
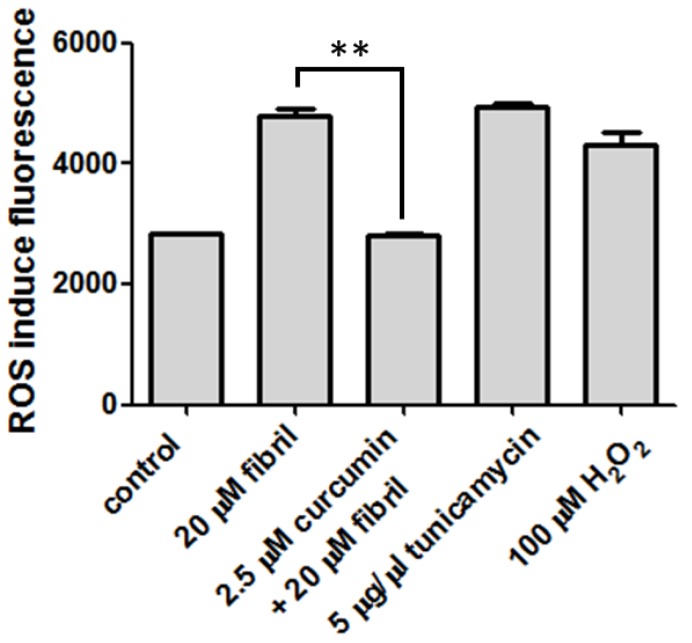
ROS level of N2a cells determined by intracellular ROS assay. The statistical significance is represented by two asterisks (*p* < 0.01).

Previous experiments with mice have hinted at the involvement of PrP in signaling pathways [27]. The activity of the PrP-dependent cascade is sensitive to ROS. The production of ROS is significantly increased in the brain mitochondrial fractions of scrapie-infected mice [28]. Increase in ROS level is a characteristic of apoptosis in the middle of cell cycle. This step is emphasized when redox reactions are involved. Since curcumin is widely applied for its anti-oxidant property, it is essential to investigate the effect of curcumin on the ROS level of N2a cells infected with mPrP fibrils. To determine the ROS level in N2a cells, the level of highly fluorescent DCF released when DCFH-DA is de-esterified intracellularly upon oxidation was monitored. As compared in Figure 7, co-incubation with 20 μM of mPrP fibrils significantly increased the ROS level from 2800 to 4800. The two-fold increase of ROS level is comparable to those observed for two positives, 5 μg/μL of tunicamycin and 100 μM of H_2_O_2_. The mPrP fibril-induced ROS can be abolished completely by treatment of 2.5 μM of curcumin prior to co-incubation with mPrP fibrils. The complete eradication of ROS caused by mPrP fibrils suggests that curcumin is involved in the ROS-related signal transduction pathways. The involvement of ROS-sensitive transcription factor NF-κB in brain mitochondrial fractions has been reported [28]. In addition, activation of GSK-3 has been found to be a critical mediator of prion peptide-induced neurodegeneration [29]. A similar role by GSK-3 has been reported in the study of amyloid-β [25]. GSK-3 phosphorylation is an upstream event of the ROS generation and the eradication of fibril-induced ROS could be due to the inhibition of GSK-3 as in the case of amyloid-β [30].

## 3. Experimental Section

### 3.1. mPrP Expression and Purification

Plasmid pET101 encoding mPrP 23–231 was transformed into competent *E. coli* BL21 (DE3), and expressed in inclusion bodies by induction with isopropyl *β*_-D-_thiogalactopyranoside. The protein was purified on a Ni-Sepharose column according to a previously described procedure [14]. The purity of the isolated protein was confirmed by SDS–PAGE.

### 3.2. Kinetics of Fibril Conversion

The fibril conversion from 10 μM mouse prion protein (mPrP) was incubated on the shaker rotating at 1000 rpm at 37 °C in a buffer solution containing 50 mM MES (pH 6.0) and 2 M GdnHCl. In addition, tested compounds were added into the sample mixture. The kinetics of fibril formation was monitored by thioflavin T (ThT), a fluorescence dye recognizing amyloids. The aliquots of fibril samples during the time course of incubation were diluted with sodium acetate buffer (pH 5.0) containing 10 μM ThT to the final fibril concentration at 0.5 μM. The fluorescence spectra were collected from 470 to 550 nm with the excitation wavelength at 450 nm by using a Hitachi F-4500 fluorimeter. The maximum fluorescence emission at 482 nm was recorded.

### 3.3. TEM

The fibril samples were stained with 2.6% tungsten phosphoric acid on carbon-coated 200-mesh copper grids. The samples were adsorbed onto the copper grids for 1 min and subsequently washed with PBS and H_2_O. The samples were air-dried before imaging. The TEM images were collected using a Hitachi H-7100 TEM. The analysis of fibril length was performed with ImageJ software.

### 3.4. Proteinase K Digestion

The 10 μM of fibril samples were treated with proteinase K (PK) at 37 °C in 100 mM Tris (pH 7.5). After one hour incubation, the PK-treated samples were added with 2× sample buffer for SDS-PAGE followed by heating at 95 °C for 10 min and then analyzed by tricine-SDS-PAGE. The densitometric quantification of proteins was analyzed with ImageJ software.

### 3.5. Hemolytic Assay

The mouse blood was primarily centrifuged at 1,000 g for 10 min. After removing the supernatant, the erythrocytes were washed three times with phosphate buffered saline (PBS, pH7.4). Subsequently, the mPrP fibrils were added into the cell suspensions (1% hematocrit), and incubated at 37 °C for 40 min. The mixtures were centrifuged at 1,000 g for 10 min. Finally, the aliquots of cells were distinguished by microscopy and the aliquots of supernatant were collected for absorption measurement.

### 3.6. Cell Culture and Viability Assay

Mouse neuroblastoma (N2a) cells were maintained in Dulbecco’s modified Eagle’s medium (DMEM) supplemented with penicillin/streptomycin and 10% (v/v) fetal bovine serum in a humidified atmosphere with 5% CO_2_ at 37 °C. N2a cells (1 × 10^4^) were seeded to 96-well plates for 24 h, and then treated with curcumin and/or mPrP fibrils. After 72 h of incubation, the medium was removed and the cell plates were washed by PBS. Subsequently, 10% (v/v) WST-1 was added into the plates and incubated with cells for 3 h. The cell viability was determined by the absorbance of formazan was recorded at 450 nm using an ELISA reader

### 3.7. Cell Apoptosis Analysis

Cells were fixed by adding 100% methanol drop by drop then stored at 4 °C for over 30 min. The fixed cells were spin down followed by re-suspension in 500 μL of PBS. Cells were then incubated with RNase to final concentration at 200 μg/mL and with propidium iodide (PI) to final concentration at 40 μg/mL at room temperature in the dark for 30 min. The cell cycle profiles were analyzed by Cytomics FC500 Flow Cytometry (Beckman Coulter). 

### 3.8. Cell ROS Measurement

N2a cells (1 × 10^5^) were seeded to 24-well plates for 24 h, and then treated with 20 μM of mPrP fibrils, or 2.5 μM curcumin prior to co-incubation of mPrP fibrils. Two positive controls including 5 μg/μL of tunicamycin and 100 μM H_2_O_2_ were compared. After 2 h of incubation, 2, 7-dichrofluorescein diacetate (DCFH-DA) was added to the cell plates to final concentration of 50 μM, and then incubated for 30 min. Subsequently, the medium was removed and the cell plates were washed by PBS, followed by the addition of 1 mM N-acetylcysteine dissolved in DMSO to quench the oxidation of DCFH-DA. The fluorescence emission of reduced 2, 7-dichrofluorescein (DCF) at 530 nm was recorded with the excitation wavelength at 485 nm by using a fluorescence ELISA reader.

## 4. Conclusions

In the current study, we verified the inhibition of amyloid formation from prion by curcumin. We also found that a low dose of curcumin can effectively rescue cells from amyloid-induced apoptosis and reactive oxidative stress. The daily intake of curcumin may potentially prevent transmissible neurodegenerative diseases, but more clinical trials are required to validate the effect of dietary curcumin on the prevention of neurodegenerative diseases.
